# Year in review 2013: *Critical Care* - metabolism

**DOI:** 10.1186/s13054-014-0571-4

**Published:** 2014-10-27

**Authors:** Olivier Lheureux, Jean-Charles Preiser

**Affiliations:** Department of Intensive Care, Erasmus University Hospital, 808 route de Lennik, B1070 Brussels, Belgium

## Abstract

Novel insights into the metabolic alterations of critical illness, including new findings on association between blood glucose at admission and poor outcome, were published in *Critical Care* in 2013. The role of diabetic status in the relation of the three domains of glycemic control (hyperglycemia, hypoglycemia, and glycemic variability) was clarified: the association between mean glucose, high glucose variability, and ICU mortality was stronger in the non-diabetic than in diabetic patients. Improvements in the understanding of pathophysiological mechanisms of stress hyperglycemia were presented. Novel developments for the management of glucose control included automated closed-loop algorithms based on subcutaneous glucose measurements and microdialysis techniques. In the field of obesity, some new hypotheses that could explain the ‘obesity paradox’ were released, and a role of adipose tissue in the response to stress was suggested by the time course of adipocyte fatty-acid binding protein concentrations. In the field of nutrition, beneficial immunological effects have been associated with early enteral nutrition. Early enteral nutrition was significantly associated with potential beneficial effects on the phenotype of lymphocytes. Uncertainties regarding the potential benefits of small intestine feeding compared with gastric feeding were further investigated. No significant differences were observed between the nasogastric and nasojejunal feeding groups in the incidence of mortality, tracheal aspiration, or exacerbation of pain. The major risk factors to develop diarrhea in the ICU were described. Finally, the understanding of disorders associated with trauma and potential benefits of blood acidification was improved by new experimental findings.

## Introduction

In *Critical Care* in 2013, several important contributions in the fields of physiology and clinical management of glucose control, enteral nutrition, and gastrointestinal disorders; specificity of critically ill obese patients; and critical illness-related endocrine alterations were published. These articles can be gathered into four areas of interest.

## Glucose metabolism and control

The management of stress-related hyperglycemia has been the focus of several prospective studies that yielded results still fueling a hot debate [1,2]. The concept of the three domains of dysglycemia (hyperglycemia, hypoglycemia, and glycemic variability) has emerged over the last year. Indeed, all three domains have been independently associated with increased risk of mortality in ICU patients [3,4]. Analysis of the results showed that hypoglycemia had the strongest association with mortality and that the negative effects of hyperglycemia and greater glycemic variability were additive. Nowadays, the magnitude of stress-related hyperglycemia can be considered a surrogate marker of the severity of disease. Hence, an optimal target of glucose control is probably elusive, and the quality of glucose control should rather be considered a quality indicator of critical care service. Those findings fostered research in several different fields. Epidemiological insights included association studies between blood glucose (BG) and outcome, endocrine pathways were investigated as potential contributors of stress hyperglycemia, and computer-assisted decision systems and continuous glucose monitoring were assessed in clinical conditions.

### Association between blood glucose levels and outcome

Recently, admission BG level was demonstrated as an independent predictor of mortality in patients with ST-segment elevation myocardial infarction (STEMI) regardless of the diabetic status [5]. Between November 2005 and September 2010, 816 STEMI patients with cardiogenic shock were enrolled in a prospective multicenter Korean study that investigated the impact of admission BG on 30-day mortality [6]. The 30-day mortality rates were higher in patients with higher BG levels in non-diabetic patients but not in diabetic patients, suggesting that the toxic effect of hyperglycemia may be limited in patients with known diabetes mellitus (DM).

The influence of pre-existing DM on the relation of these three domains of glycemic control with mortality remained uncertain. A new study examining markers of glycemic control in a DM cohort and a non-DM cohort simultaneously was published last year [7]. This single-center retrospective study performed on a cohort of more than 10,320 patients (of whom 16% had DM) reported a strong association between mean glucose and high glucose variability and ICU mortality in the non-DM cohort only. Hypoglycemia (≤2.2 mmol/L) was associated with ICU mortality in both cohorts. Secondly, a higher threshold for toxicity was found in the non-DM cohort (4.9 mmol/L) than in the DM cohort (3.5 mmol/L), suggesting once again that DM patients may tolerate a wider glucose range. This could be explained by the fact that patients with DM may develop a cellular adaption over time as the reduction of mitochondrial-derived reactive oxygen species and therefore better tolerate episodes of hyperglycemia in an acute care setting [8,9].

### Physiopathology of stress hyperglycemia

The metabolic response to stress is part of the adaptive response to survive critical illness. It involves a subsequent neuroendocrine and an immune component leading to an uncontrolled catabolism, the development of resistance to anabolic signals (including insulin), and an inability to suppress the central hepatic glucose production [10]. Many acute metabolic changes seen in critically ill patients are similar to those seen in patients with metabolic syndrome. Similar changes can be involved in the acute stress responses associated with critical illness [11]. Adipocyte fatty-acid binding protein (A-FABP) is one of the most abundant intracellular lipid transport proteins in mature adipocytes and macrophages. Recent studies have confirmed its role in various conditions associated with insulin resistance, including metabolic syndrome [12,13]. More than 100 patients admitted to a medical ICU were prospectively enrolled in a study whose purpose was to determine the correlation between A-FABP, systemic inflammation, and insulin resistance associated with critical illness [14]. Blood samples were collected within 48 hours of ICU admission. Elevated A-FABP concentrations in patients with critical illness were positively correlated with Acute Physiology and Chronic Health Evaluation II (APACHE II) scores, inflammatory cytokine tumor necrosis factor-alpha, and insulin resistance. The A-FABP concentrations were not associated with pre-existing DM and body mass index (BMI), suggesting that circulating A-FABP reflects critical illness-related insulin resistance. This major finding suggests a major role for the adipose in the response to stress.

### Clinical management of glucose control

Accurate measurements are critical for treatment decisions. Actually, repeated blood gas analyses represent the best option to measure BG within the ICU [15]. A systematic review assessed the accuracy of BG measurements in the ICU by using different techniques (by glucose meters and arterial blood gas analyzers) with central laboratory methods as references [16]. Results of the study showed a significantly higher accuracy of BG measurements with arterial gas analyzers than measurements with glucose meters. These data also suggest that arterial blood samples rather than capillary blood samples should be used and certainly in patients with unstable hemodynamics or receiving insulin infusion or both. Otherwise, BG measurements in the hypoglycemic range were less accurate than those in the non-hypoglycemic range in all devices.

New technologies could improve the management of BG and help to prevent the three domains of dysglycemia.

Over the last decade, continuous subcutaneous glucose monitoring has emerged as a valuable tool in the management of diabetes [17]. A pilot preliminary randomized controlled single-center trial evaluated the feasibility of automated closed-loop glucose control based on subcutaneous glucose measurements in critically ill adults [18]. Twenty-four patients hospitalized in a neurologic ICU with hyperglycemia (≥10 mM) or already receiving insulin therapy were randomly assigned to receive one of the following over a 48-hour period: fully automated closed-loop therapy (model predictive control algorithm directing insulin and 20% dextrose infusion based on FreeStyle Navigator (Abbott Laboratories, Abbott Park, IL, USA) continuous subcutaneous glucose values, n =12) or a local protocol (moderate glucose target of 6.0 to 8.0 mmol/L, n =12) with intravenous sliding-scale insulin. The primary endpoint was percentage of time when arterial blood glucose was between 6.0 and 8.0 mmol/L. The proportion of time in target range was significantly increased during closed-loop therapy (54.3% versus 18.5%, *P* =0.001). Mean glucose was significantly lower during closed-loop without hypoglycemia (<4 mmol/L) during either therapy. No adverse event linked to the sensor was recorded. These results suggested that fully automated closed-loop control based on subcutaneous glucose measurements might provide safe, effective, and consistent glucose control without increasing the risk of hypoglycemia.

A pilot observational prospective study tested a central vein catheter with a microdialysis membrane in combination with an on-line analyzer for continuous monitoring of circulating glucose and lactate by the central route [19]. Ten patients scheduled for major upper abdominal surgery were enrolled and received an extra central venous catheter (Eirus SLC, Dipylon Medical AB, Solna, Sweden). Continuous microdialysis measurement proceeded for 20 hours, and on-line values were collected every minute. Reference arterial plasma glucose and blood lactate samples were collected every hour. Results showed a close agreement between the continuous reading and the reference BG values and a high correlation to plasma readings (r =0.92). The intravascular microdialysis technique using central venous access shows promising results compared with reference plasma values, justifying further testing, especially in ICU patients.

## Outcome of obese patients with sepsis

Obesity is an increasingly prevalent comorbidity in critically ill patients. Despite the apparent risk factors associated with obesity, several studies suggested better outcome for obese than non-obese patients [20]. This phenomenon has been named the ‘obesity paradox’. Several studies focused on the subject last year.

Firstly, a retrospective analysis compared three groups of septic shock patients on the basis of the BMI in patients enrolled in the VASST (Vasopressin and Septic Shock Trial) (n =778 patients) cohort [21]. Obese (BMI of greater than 30 kg/m^2^, n =245) and overweight (BMI of 25.0 to 29.9 kg/m^2^, n =276) patients with septic shock had significantly lower 28-day mortality and less organ dysfunction than patients with a BMI of less than 25 kg/m^2^, despite similar severity at presentation. Compared with the patients with a BMI of less than 25 kg/m^2^, obese and overweight patients also had a different pattern of infection with less lung and fungal infection. Per kilogram, obese and overweight patients received less fluid and less norepinephrine or vasopressin compared with patients with a BMI of less than 25 kg/m^2^. Obese and overweight patients also had lower plasma interleukin-6 concentration at baseline. However, the reason why these patients present an altered inflammatory response is still unknown.

Secondly, a nested cohort study (n =2,882) within a multicenter retrospective database of patients with septic shock has been published [22]. Very obese patients more frequently developed skin and soft tissue infections and were less likely to have pneumonia. Obese and very obese patients were more likely to have Gram-positive infections. These patients received a lower amount of resuscitation fluids and dose of antibiotics per kilogram. Similarly to the previous study, obese and very obese patients had significantly lower mortality compared with normal-weight patients. However, this ‘obesity paradox’ may be explained in part by differences in baseline characteristics and sepsis interventions, especially the volume in resuscitation. Indeed, very obese patients with septic shock had fewer hemodynamic disturbances and required lower doses of vasopressors than normal-weight patients, though with comparable APACHE II scores.

## Enteral nutrition and gastrointestinal disorders

### Early versus late enteral nutrition

Compared with parenteral nutrition support, the introduction of early enteral nutrition within the first 24 to 48 hours after ICU admission has been associated with positive effects: less septic complications, better course of primary disease, and shorter stay in the ICU [23]. These findings were attributed to the prevention of worsening in intestinal permeability, interruption of the catabolic process, and restoration of the immune response. Incretin hormones, like glucagon-like-peptide-1 (GLP-1) and gastric inhibitory polypeptide, originate from the gastrointestinal system in response to the presence of nutrition in the intestinal lumen and potentiate postprandial insulin secretion. GLP-1 is secreted principally from the L cells of the distal ileum. In a recent study, GLP-1 was shown to act as an immune modulator and influence cell-mediated immunity [24]. The impacts of early enteral nutrition and late enteral nutrition (beginning 48 hours after admission) on plasma GLP-1 levels of 20 ICU patients with thromboembolic stroke were determined in a clinical trial [25]. Daily caloric requirement was determined as 25 kcal/kg per day for each patient according to the European Society of Parenteral and Enteral Nutrition [26]. No significant difference in pre-/post-feeding GLP-1 levels was observed between groups. However, early enteral nutrition was significantly associated with increased numbers of T helper and regulatory T cells and decreased amounts of T cytotoxic cells without any change in plasma GLP-1, suggesting enteral nutrition-induced effects on the phenotype of lymphocytes.

### Feeding routes

Previous studies demonstrated that early enteral nutrition reduces the risk of infections when compared with parenteral nutrition [27]. Whether the feeding tubes should be preferentially placed into the stomach or small bowel remains contentious. Nasogastric tubes are relatively easy to insert. However, the disadvantage of the intragastric approach includes delayed gastric emptying and predisposes to inadequate nutrient administration. Small bowel feeding tubes are more difficult to insert, often requiring specific expertise and equipment. Their potential advantages include bypassing the stomach, which should theoretically guarantee delivery of nutrients. In fact, the major gastrointestinal motility disorders in the critically ill appear to occur in the antral-pyloro region of the stomach. A further consideration is that the delivery of nutrients into the small bowel does imply an intact absorption of nutrients.

Over the past decade, four systematic reviews were published about the risk of pneumonia with gastric or small bowel feeding and seemingly reached conflicting results. Two suggested that small bowel feeding reduces the risk of pneumonia [28,29], whereas the other two did not [30,31]. These conflicting results could be related to differences in search strategies, type of patients included, management of gastric emptying, or outcome definition. A new systematic review and meta-analysis including 19 trials (n =1,394 patients) tried to determine the effect of small bowel feeding compared with gastric feeding on the frequency of pneumonia [32]. The results showed that small bowel feeding in comparison with gastric feeding reduces the risk of pneumonia in critically ill patients, but no difference was seen in mortality, length of ICU stay, or duration of mechanical ventilation. The mechanism by which small bowel feeding could reduce pneumonia is not entirely clear. It has been presumed that increased gastric volume leads to regurgitation and aspiration, yet multiple studies failed to demonstrate an association between elevated gastric residual volume and risk of aspiration [33]. A second systematic review of the same topic was published last year [34]. All randomized controlled studies published between 1990 and 2013 were included. As in the previous study, small bowel feeding was associated with a reduced risk of pneumonia. Duration of ventilation and mortality were also unaffected by the route of feeding.

As recommended by current guidelines [35], nutritional support using enteral nutrition should be the preferred method in patients with severe acute pancreatitis. A meta-analysis, which aimed to determine an ideal nutrient feeding approach, included three randomized controlled trials (n =157 patients) comparing nasogastric and nasojejunal feeding in patients with predicted severe acute pancreatitis [36]. No significant differences were observed between the two groups in the incidence of mortality, tracheal aspiration, or exacerbation of pain. Also, the achievement of energy balance was not different, suggesting that nasogastric feeding is safe and well tolerated compared with nasojejunal feeding. All together, patients with relatively ‘normal’ gastric emptying and esophago-gastric motility are unlikely to benefit from small intestinal feeding.

### Assessment of whole-body protein turnover

The measurement of protein turnover, including the rate of protein synthesis and degradation, is challenging during critical illness. A pilot study included 16 neurosurgical patients to evaluate the effect of low and high caloric intake on whole-body protein turnover assessed by radiolabeled leucine and phenylalanine [37]. Overall, low caloric intake was associated with a more negative protein balance compared with high caloric intake. However, the rate of oxidation of amino acids was unchanged by the caloric load. This study demonstrates that a sophisticated assessment of whole-body protein turnover is feasible and helpful to improve the understanding of the metabolic response to stress of critically ill patients.

### Diarrhea

Conflicting results were obtained from previous trials about the impact of enteral nutrition on the occurrence of diarrhea. Some authors argued that enteral nutrition reduces the incidence of diarrhea through a better preservation of intestinal mucosa, whereas others found a positive relation between enteral nutrition and diarrhea [38]. The incidence and risk factors for diarrhea during the 14 first days of ICU stay were determined in a prospective observational study (n =278 patients) in a tertiary ICU population [39]. Diarrhea was observed in 38 patients (14%). *Clostridium difficile* infection is always suspected when diarrhea occurs in the ICU, mainly in patients with antibiotics, but a low incidence of *C. difficile* was observed (0.7%), which is in accordance with previous studies [40]. The presence of enteral nutrition *per se* had no impact on the risk of diarrhea. However, enteral nutrition, when delivering more than 60% of the energy target, increased the risk of diarrhea (relative risk =1.75). The other factors significantly and independently associated with the risk of diarrhea were antibiotics (relative risk =3.64) and antifungal drugs (relative risk =2.79). Furthermore, when combined, these negative effects were additive. These results suggest that enteral nutrition, even if covering the energy target, should not be incriminated as the sole cause of diarrhea in ICU. Because other factors could increase the diarrhea risk, diarrhea onset in ICU patients receiving enteral nutrition must not be systematically considered a non-functionality of the gastrointestinal tract and should not lead to systematic discontinuation of enteral nutrition. Otherwise, if enteral nutrition is considered the primary cause of diarrhea, changes in the administration flow rate or replacement of the enteral nutrition solution can be considered.

## Experimental studies

Three animal studies that focused on different topics were published last year. The first study evaluated the two major components that accompany severe trauma: a period of hypermetabolism and disuse [41]. These two combined components contribute to a multitude of issues limiting or prolonging (or both) recovery from injury. Therefore, a rat model combining immobilization and severe burn was used to determine the effects of burn and disuse, independently and in combination, on body composition, food intake, and adipokines (leptin, resistin, and adiponectin) that have been associated with the inflammatory response to injury, insulin resistance, and the severity of illness [42,43]. The results of the study showed that, independently, burn injury and disuse have similar body mass reductions from control. However, when combined, additive effects were apparent. Reduced adipokine levels were observed in the presence of injury rather than disuse, suggesting a greater influence of the injury component. Furthermore, the observed changes in adipokines provide insight for intervention to attenuate the hypermetabolic state following injury.

The second study assessed, in an ovine model, the impact of lactic acid infusion on whole-body CO_2_ production compared with an isocaloric glucose infusion [44]. In case of partial extracorporeal CO_2_ removal, the rate of CO_2_ removal is limited by the fact that most of the CO_2_ in blood is present as bicarbonate ion that cannot cross the artificial lung membrane. In those circumstances, infusion of lactic acid may be beneficial [45]. Indeed, acid infusion shifts bicarbonate dissociation to the gaseous CO_2_ form, increasing the transmembrane pressure gradient and thus increasing extracorporeal CO_2_ removal. However, lactic acid metabolism might increase total body CO_2_ production, limiting the potential beneficial effects of this technique. A slight increase in CO_2_ production (less than 5%) was observed after the administration of 50% of the total caloric input with an infusion of lactic acid compared with an equal caloric load provided entirely by infusion of 50% glucose solution. Blood acidification at the inlet of a membrane lung therefore should be considered a promising technique to reduce the ventilator needs.

## Conclusions

The areas of critical illness-associated metabolic and endocrine alterations received increasingly large attention reflected by the articles published in 2013. The issues of stress hyperglycemia and glucose control, including on-line/continuous glucose measurement techniques, were further investigated. The ‘obesity paradox’ phenomenon has been a particularly hot topic. The nutritional aspects of critical illness draw attention to the benefits of early enteral nutrition, especially its immune consequences. All together, new fields of research were opened by the high-quality articles published in *Critical Care* in 2013.

## Note

This article is part of a collection of Year in review articles in *Critical Care*. Other articles in this series can be found at [46].

## Abbreviations

A-FABP: Adipocyte fatty-acid binding protein
APACHE II: Acute Physiology and Chronic Health Evaluation II
BG: Blood glucose
BMI: Body mass index
DM: Diabetes mellitus
GLP-1: Glucagon-like-peptide-1
STEMI: ST-segment elevation myocardial infarction

## References

[1] Marik PE, Preiser JC (2010). Toward understanding tight glycemic control in the ICU: a systematic review and metaanalysis. Chest.

[2] Van den Berghe G (2012). Intensive insulin therapy in the ICU - reconciling the evidence. Nat Rev Endocrinol.

[3] Krinsley JS (2011). Understanding glycemic control in the critically ill: three domains are better than one. Intensive Care Med.

[4] Mackenzie IM, Whitehouse T, Nightingale PG (2011). The metrics of glycaemic control in critical care. Intensive Care Med.

[5] Hoebers LP, Damman P, Claessen BE, Vis MM, Baan J, van Straalen JP, Fischer J, Koch KT, Tijssen JG, de Winter RJ, Piek JJ, Henriques JP (2012). Predictive value of plasma glucose level on admission for short and long term mortality in patients with ST-elevation myocardial infarction treated with primary percutaneous coronary intervention. Am J Cardiol.

[6] Yang JH, Song PS, Song YB, Hahn JY, Choi SH, Choi JH, Lee SH, Jeong MH, Kim YJ, Gwon HC (2013). Prognostic value of admission blood glucose level in patients with and without diabetes mellitus who sustain ST segment elevation myocardial infarction complicated by cardiogenic shock. Crit Care.

[7] Sechterberger MK, Bosman RJ, Oudemans-van Straaten HM, Siegelaar SE, Hermanides J, Hoekstra JB, De Vries JH (2013). The effect of diabetes mellitus on the association between measures of glycaemic control and ICU mortality: a retrospective cohort study. Crit Care.

[8] Dugan LL, You YH, Ali SS, Diamond-Stanic M, Miyamoto S, DeCleves AE, Andreyev A, Quach T, Ly S, Shekhtman G, Nguyen W, Chepetan A, Le TP, Wang L, Xu M, Paik KP, Fogo A, Viollet B, Murphy A, Brosius F, Naviaux RK, Sharma K (2013). AMPK dysregulation promotes diabetes-related reduction of superoxide and mitochondrial function. J Clin Invest.

[9] Brownlee M (2001). Biochemistry and molecular cell biology of diabetic complications. Nature.

[10] Dungan KM, Braithwaite SS, Preiser JC (2009). Stress hyperglycaemia. Lancet.

[11] Bagry HS, Raghavendran S, Carli F (2008). Metabolic syndrome and insulin resistance: perioperative considerations. Anesthesiology.

[12] Xu A, Tso AW, Cheung BM, Wang Y, Wat NM, Fong CH, Yeung DC, Janus ED, Sham PC, Lam KS (2007). Circulating adipocyte-fatty acid binding protein levels predict the development of the metabolic syndrome: a 5-year prospective study. Circulation.

[13] Toruner F, Altinova AE, Akturk M, Kaya M, Arslan E, Bukan N, Kan E, Yetkin I, Arslan M (2011). The relationship between adipocyte fatty acid binding protein-4, retinol binding protein-4 levels and early diabetic nephropathy in patients with type 2 diabetes. Diabetes Res Clin Pract.

[14] Huang C-L, Wu Y-W, Hsieh A-R, Hung Y-H, Chen W-J, Yang W-S (2013). Serum adipocyte fatty acid-binding protein levels in patients with critical illness are associated with insulin resistance and predict mortality. Crit Care.

[15] Finfer S, Wernerman J, Preiser JC, Cass T, Desaive T, Hovorka R, Joseph JI, Kosiborod M, Krinsley J, Mackenzie I, Mesotten D, Schultz MJ, Scott MG, Slingerland R, Van den Berghe G, Van Herpe T (2013). Clinical review: consensus recommendations on measurement of blood glucose and reporting glycemic control in critically ill adults. Crit Care.

[16] Inoue S, Egi M, Kotani J, Morita K (2013). Accuracy of blood-glucose measurements using glucose meters and arterial blood gas analyzers in critically ill adult patients: systematic review. Crit Care.

[17] Pickup JC, Freeman SC, Sutton AJ (2011). Glycaemic control in type 1 diabetes during real time continuous glucose monitoring compared with self monitoring of blood glucose: meta-analysis of randomised controlled trials using individual patient data. BMJ.

[18] Leelarathna L, English SW, Thabit H, Caldwell K, Allen JM, Kumareswaran K, Wilinska ME, Nodale M, Mangat J, Evans ML, Burnstein R, Hovorka R (2013). Feasibility of fully automated closed-loop glucose control using continuous subcutaneous glucose measurements in critical illness: a randomized controlled trial. Crit Care.

[19] Blixt C, Rooyackers O, Isaksson B, Wernerman J (2013). Continuous on-line glucose measurement by microdialysis in a central vein. A Pilot Study Crit Care.

[20] Habbu A, Lakkis NM, Dokainish H (2006). The obesity paradox: fact or fiction?. Am J Cardiol.

[21] Wacharasint P, Boyd JH, Russell JA, Walley KR (2013). One size does not fit all in severe infection: obesity alters outcome, susceptibility, treatment, and inflammatory response. Crit Care.

[22] Arabi YM, Dara SI, Tamim HM, Rishu AH, Bouchama A, Khedr MK, Feinstein D, Parrillo JE, Wood KE, Keenan SP, Zanotti S, Martinka G, Kumar A, Kumar A, The Cooperative Antimicrobial Therapy of Septic Shock (CATSS) Database Research Group (2013). Clinical characteristics, sepsis interventions and outcomes in the obese patients with septic shock: an international multicenter cohort study. Crit Care.

[23] McClave SA, Martindale RG, Vanek VW, McCarthy M, Roberts P, Taylor B, Ochoa JB, Napolitano L, Cresci G, A.S.P.E.N. Board of Directors; American College of Critical Care Medicine; Society of Critical Care Medicine (2009). Guidelines for the Provision and Assessment of Nutrition Support Therapy in the Adult Critically Ill Patient: Society of Critical Care Medicine (SCCM) and American Society for Parenteral and Enteral Nutrition (A.S.P.E.N.). JPEN J Parenter Enteral Nutr.

[24] Kim SJ, Nian C, Doudet DJ, McIntosh CH (2009). Dipeptidyl peptidase IV inhibition with MK0431 improves islet graft survival in diabetic NOD mice partially via T-cell modulation. Diabetes.

[25] Bakiner O, Bozkirli E, Giray S, Arlier Z, Kozanoglu I, Sezgin N, Sariturk C, Ertorer E (2013). Impact of early versus late enteral nutrition on cell mediated immunity and its relationship with glucagon like peptide-1 in intensive care unit patients: a prospective study. Crit Care.

[26] Kreymann KG, Berger MM, Deutz NE, Hiesmayr M, Jolliet P, Kazandjiev G, Nitenberg G, van den Berghe G, Wernerman J, Ebner C, Hartl W, Heymann C, Spies C, DGEM (German Society for Nutritional Medicine), ESPEN (European Society for Parenteral and Enteral Nutrition) (2006). ESPEN Guidelines on Enteral Nutrition: Intensive care. Clin Nutr.

[27] Gramlich L, Kichian K, Pinilla J, Rodych NJ, Dhaliwal R, Heyland DK (2004). Does enteral nutrition compared to parenteral nutrition result in better outcomes in critically ill adult patients? A systematic review of the literature. Nutrition.

[28] Heyland DK, Drover JW, Dhaliwal R, Greenwood J (2002). Optimizing the benefits and minimizing the risks of enteral nutrition in the critically ill: role of small bowel feeding. JPEN J Parenter Enteral Nutr.

[29] Jiyong J, Tiancha H, Huiqin W, Jingfen J (2013). Effect of gastric versus post-pyloric feeding on the incidence of pneumonia in critically ill patients: observations from traditional and Bayesian random-effects meta-analysis. Clin Nutr.

[30] Marik PE, Zaloga GP (2003). Gastric versus post-pyloric feeding: a systematic review. Crit Care.

[31] Ho KM, Dobb GJ, Webb SA (2006). A comparison of early gastric and post-pyloric feeding in critically ill patients: a meta-analysis. Intensive Care Med.

[32] Alhazzani W, Almasoud A, Jaeschke R, Lo BW Y, Sindi A, Altayyar S, Fox-Robichaud AE (2013). Small bowel feeding and risk of pneumonia in adult critically ill patients: a systematic review and meta-analysis of randomized trials. Crit Care.

[33] Reignier J, Mercier E, Le Gouge A, Boulain T, Desachy A, Bellec F, Clavel M, Frat JP, Plantefeve G, Quenot JP, Lascarrou JB, Clinical Research in Intensive Care and Sepsis (CRICS) Group (2013). Effect of not monitoring residual gastric volume on risk of ventilator-associated pneumonia in adults receiving mechanical ventilation and early enteral feeding: a randomized controlled trial. JAMA.

[34] Deane AM, Rupinder D, Day AG, Ridley EJ, Davies AR, Heyland DK (2013). Comparisons between intragastric and small intestinal delivery of enteral nutrition in the critically ill: a systematic review and meta-analysis. Crit Care.

[35] Mirtallo JM, Forbes A, McClave SA, Jensen GL, Waitzberg DL, Davies AR (2012). International consensus guidelines for nutrition therapy in pancreatitis. JPEN J Parenter Enteral Nutr.

[36] Chang Y-S, Fu H-Q, Xiao Y-M, Liu J-C (2013). Nasogastric or nasojejunal feeding in predicted severe acute pancreatitis: a meta-analysis. Crit Care.

[37] Berg A, Rooyackers O, Bellander B-M, Wernerman J (2013). Whole body protein kinetics during hypocaloric and normocaloric feeding in critically ill patients. Crit Care.

[38] Wiesen P, Van Gossum A, Preiser JC (2006). Diarrhoea in the critically ill. Curr Opin Crit Care.

[39] Thibault R, Graf S, Clerc A, Delieuvin N, Heidegger CP, Pichard C (2013). Diarrhoea in the ICU: respective contribution of feeding and antibiotics. Crit Care.

[40] Nguyen NQ, Ching K, Fraser RJ, Chapman MJ, Holloway RH (2008). Risk of Clostridium difficile diarrhoea in critically ill patients treated with erythromycin-based prokinetic therapy for feed intolerance. Intensive Care Med.

[41] Wade CE, Baer LA, Wu X, Silliman DT, Walters TJ, Wolf SE (2013). Severe burn and disuse in the rat independently adversely impact body composition and adipokines. Crit Care.

[42] Langouche L, Vander Perre S, Frystyk J, Flyvbjerg A, Hansen TK, Van den Berghe G (2009). Adiponectin, retinol-binding protein 4, and leptin in protracted critical illness of pulmonary origin. Crit Care.

[43] Venkatesh B, Hickman I, Nisbet J, Cohen J, Prins J (2009). Changes in serum adiponectin concentrations in critical illness: a preliminary investigation. Crit Care.

[44] Zanella A, Giani M, Redaelli S, Mangili P, Scaravilli V, Ormas V, Costanzi M, Albertini M, Bellani G, Patroniti N, Pesenti A (2013). Infusion of 2.5 meq/min of lactic acid minimally increases CO2 production compared to an isocaloric glucose infusion in healthy anesthetized, mechanically ventilated pigs. Crit Care.

[45] Zanella A, Patroniti N, Isgrò S, Albertini M, Costanzi M, Pirrone F, Scaravilli V, Vergnano B, Pesenti A (2009). Blood acidification enhances carbon dioxide removal of membrane lung: an experimental study. Intensive Care Med.

[46] http://ccforum.com/series/Yearinreview2013.

